# The Oral Microbiome and Cancer

**DOI:** 10.3389/fimmu.2020.591088

**Published:** 2020-10-23

**Authors:** Muhammad Irfan, Renata Zoraida Rizental Delgado, Jorge Frias-Lopez

**Affiliations:** Department of Oral Biology, College of Dentistry, University of Florida, Gainesville, FL, United States

**Keywords:** oral microbiome, cancer, oral squamous cell carcinoma, *Fusobacterium nucleatum*, *Porphyromonas gingivalis*, immunotherapy, bacteria-mediated tumor therapy

## Abstract

There is mounting evidence that members of the human microbiome are highly associated with a wide variety of cancer types. Among oral cancers, oral squamous cell carcinoma (OSCC) is the most prevalent and most commonly studied, and it is the most common malignancy of the head and neck worldwide. However, there is a void regarding the role that the oral microbiome may play in OSCC. Previous studies have not consistently found a characteristic oral microbiome composition associated with OSCC. Although a direct causality has not been proven, individual members of the oral microbiome are capable of promoting various tumorigenic functions related to cancer development. Two prominent oral pathogens, *Porphyromonas gingivalis*, and *Fusobacterium nucleatum* can promote tumor progression in mice. *P. gingivalis* infection has been associated with oro-digestive cancer, increased oral cancer invasion, and proliferation of oral cancer stem cells. The microbiome can influence the evolution of the disease by directly interacting with the human body and significantly altering the response and toxicity to various forms of cancer therapy. Recent studies have shown an association of certain phylogenetic groups with the immunotherapy treatment outcomes of certain tumors. On the other side of the coin, recently it has been a resurgence in interest on the potential use of bacteria to cure cancer. These kinds of treatments were used in the late nineteenth and early twentieth centuries as the first line of defense against cancer in some hospitals but later displaced by other types of treatments such as radiotherapy. Currently, organisms such as *Salmonella typhimurium* and *Clostridium* spp. have been used for targeted strategies as potential vectors to treat cancer. In this review, we briefly summarize our current knowledge of the role of the oral microbiome, focusing on its bacterial fraction, in cancer in general and in OSCC more precisely, and a brief description of the potential use of bacteria to target tumors.

## The Human Microbiome and Cancer. An Overview

About 30 trillion bacterial cells are living in or on every human. That is around one bacterium for each cell in the human body (1). These microorganisms are on the whole known as the microbiome. Since the completion of the Human Microbiome Project (2), we have witnessed an increased interest in the role that the human microbiome plays in human health, many studies have linked changes in microbial communities to systemic conditions such as allergies, diabetes, inflammatory bowel disease, and atherosclerosis (3–7). Among the systemic conditions influenced by the microbiome, cancer has not been an exception. We have learned that chronic infections contribute to carcinogenesis, with approximately 13% of the global cancer burden being directly attributable to infectious agents (8).

**Figure 1:**
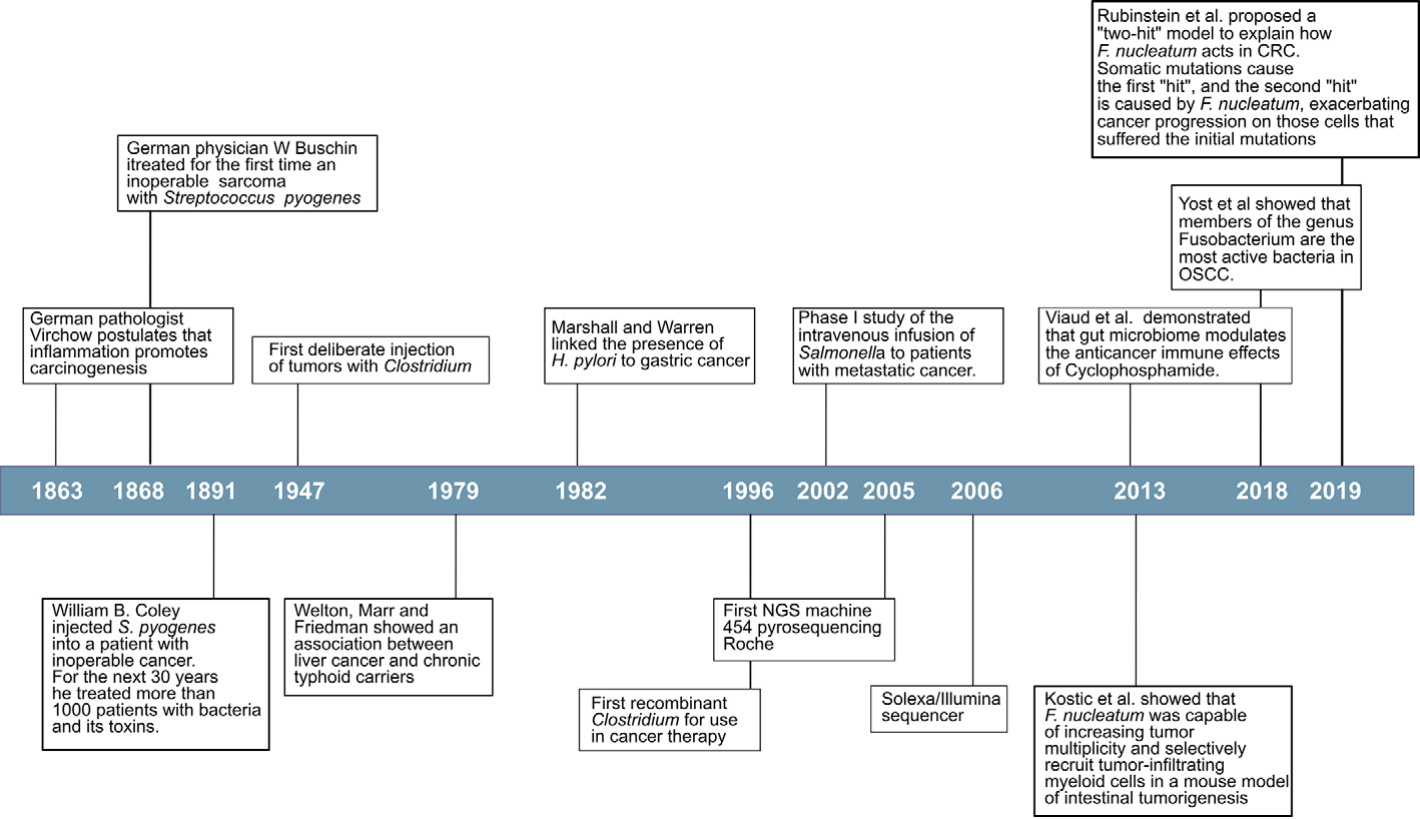
Timeline: Some significant discoveries and events in cancer microbiome research.

Many viruses promote cancer through well-described genetic mechanisms. Around 10–15% of human cancers worldwide are caused by seven human viruses, which include Epstein-Bar Virus (EBV), Hepatitis B Virus (HBV), Human T-lymphotropic virus-I (HTLV-I), Human papillomaviruses (HPV), Hepatitis C virus (HCV), Kaposi’s sarcoma herpesvirus (KSHV) and Merkel cell polyomavirus (MCV) (9). However, the first evidence that bacteria were directly involved in cancer development did not come until the 1980s with the work of Marshall and Warren (10). When they presented their results, entrenched was the belief that lifestyle caused ulcers that it was difficult for them to convince the scientific world of *Helicobacter pylori*’s role in gastric cancer (**Figure 1**). To provide even more conclusive evidence, in 1985, Marshall deliberately infected himself with the bacterium and established his stomach illness. Since then, it has been firmly proven by many researchers worldwide that *H. pylori* cause more than 90% of duodenal ulcers and up to 80% of gastric ulcers, and has been classified as a class I carcinogen by the World Health Organization due to its ability to promote stomach cancer after chronic infection (11–13). Disease-promoting and cancer-promoting effects of pathogens often depend on virulence factors. In *H. pylori*, strains expressing the virulence factors cytotoxin associated gene A (CagA) or vacuolating cytotoxin A (VacA), exemplify the role of virulence factors by increasing inflammation, and cancer rates (14).

An emerging concept in cancer biology implicates the microbiome as an influential environmental factor modulating the carcinogenic process. The idea that inflammation promotes carcinogenesis was first postulated more than 150 years ago by the German pathologist Virchow (15). The link between chronic inflammation and cancer is now well established (16–18). This association has recently experienced a renewed interest with the recognition that members of the human microbiome can be responsible for the chronic inflammation observed in a wide variety of cancers (19). Increasing evidence shows the association of changes in the human microbiome with certain types of cancer (20–22). Studies in germ-free animals have revealed evidence for tumor-promoting effects of the microbiome in spontaneous, genetically-induced, and carcinogen-induced cancers in various organs (23).

There is strong epidemiological evidence that other bacterial species are associated with cancer development, most likely induced by creating a pro-inflammatory micro-environment (24) or suppressing the immune response (25). Among the species of bacteria that have been directly linked to the development of cancer *is Salmonella enterica* subsp*. enterica* sv. *Typhi* (*S. Typhi*) and gallbladder cancer (26–28), *Streptococcus bovis* and colon cancer (29–31), and *Chlamydia pneumoniae* with lung cancer (32–36). The most persuasive epidemiological evidence of bacterial oncogenic potential, aside from *H. pylori*, concerns *S. Typhi*. However, the evidence for *S. bovis* and *C. pneumoniae* and *Fusobacterium nucleatum* is less conclusive, a meta-analysis on the association of those organisms with increased risk of cancer have shown either different or weak associations (33, 37–42).

Although gallbladder carcinoma (GBC) is rare in western countries, there is a high incidence in countries with endemic *S. Typhi* infections such as South America and parts of Africa and Asia, particularly India and Pakistan (43). The first epidemiological association was found by Welton et al. in 1979. In that paper, they analyzed 471 deceased typhoid carriers, registered by the New York City Health Department between 1922 and 1975, and matched with 942 controls for sex, age at death, year of death, the borough the carrier died, and where they were born. The results show that chronic typhoid carriers die of hepatobiliary cancer six times more often than the matched controls (44). Two more recent meta-analyses confirmed these initial results. In the first study by Koshiol et al., they performed a case-control and a meta-analysis of more than 1,000 GBC cases, and in both cases, they found a positive association between *S. Typhi* and GBC (45). In the second meta-analysis by Nagaraja and Eslick, they selected 17 studies for their analysis, most of them from India and China. The highest incidence of gallbladder cancer (GBC) occurs in India, contributing to about 10% of the global GBC cases (46). When Nagaraja and Eslick performed a subgroup analysis according to region, they found a significant association between *S. Typhi* carrier state and carcinoma of the gallbladder based on detection methods of *S. Typhi* antibody levels and culture (47). A possible mechanism has been proposed that explains the link between gallbladder carcinoma and infection by *S. Typhi*. In predispose mice, organoids and tissue culture with mutations in TP53 (tumor suppressor gene), and enhanced c-MYC (oncogenes) expression translocated bacterial effector molecules SopE, SopE2, SopB (Salmonella outer proteins), and SptP (*Salmonella* protein tyrosine phosphatase), from the *Salmonella* pathogenicity island 2 (SPI-2), activate the protein kinase B (Akt), or MAPK inhibitors, prevented mouse embryonic fibroblast transformation (48).

The previous examples refer to the link between specific organisms and carcinogenesis; however, microbes that trigger transformation events in host cells are rare. It has been demonstrated that in some cases, the tumorigenic process is not the result of the activities of a specific organism but rather the result of an instability in the composition of the bacterial communities or dysbiosis, often associated with inflammatory disorders such as colitis or periodontal disease. In mouse models, it has been shown that a dysbiotic community can lead to the development of colorectal cancer (49, 50).

The shift from a eubiotic community, with low cancer risk, to a dysbiotic community, with increased cancer risk, is the result of changes in environmental conditions and associated with metabolic responses in the host that modulate the progression of cancer (51). Dysbiosis of the oral microbiome could influence cancer outcomes by different mechanisms. Two common mechanisms that could have severe implications in the development of the disease are chronic inflammation and the synthesis of metabolites that could induce mutations (52).

### The Oral Microbiome and Cancer

There were reports of a correlation between periodontitis and leukemia as early as the late 1940s early 1950s (53, 54). Since those pioneer studies, there is mounting evidence of the correlation between periodontal disease and various cancers. Several meta-analyses have confirmed the suspicion that periodontal disease should be considered as a risk factor in several types of cancers. In fact in a meta-analysis by Corbella et al., they found that a statistically significant association was found for all cancers studied, both combined and individually (digestive tract, pancreatic, prostate, breast, corpus uteri, lung, hematological, esophageal/oropharyngeal and Non-Hodgkin lymphoma) (55). Never smokers population with periodontal disease has a higher risk of developing hematopoietic and lymphatic cancers (55, 56). Pancreatic, lung, and colorectal cancers also show a positive correlation with periodontal disease (55, 57–60). In the case of breast cancer, the evidence shows a more modest positive association between periodontal disease and breast cancer (61). Finally, the most expected correlation would be with oral cancers, and indeed there is a clear positive correlation between periodontal disease and oral cancers (55, 59, 60). Edentulism has been positively correlated with pancreatic cancer (58) but not within colorectal cancer (62). Interestingly, in the study where authors did not find associations of edentulism and colorectal cancer (CRC), they also found no correlation between edentulism and periodontitis (62). One of these meta-analyses looked at not only a correlation between periodontal disease and cancer but also correlations with particular organisms (60). The results of that meta-analysis indicated that periodontal bacterial infection increased cancer incidence and was associated with poor overall survival, disease-free survival, and cancer-specific survival. Subgroup analysis indicated that the risk of cancer was associated with *Porphyromonas gingivalis* and Prevotella intermedia infection but not *Tannerella forsythia*, *Treponema denticola*, *Aggregatibacter actinomycetemcomitans*, and *F. nucleatum* infection (60).

In the case of pancreatic cancer, a potential mechanism has been proposed (57). The innate immune response to pathogenic bacteria that results in inflammation also has been linked with pancreatic carcinogenesis. Lipopolysaccharide, a component in the outer membrane of Gram-negative bacteria, such as, *P. gingivalis*, triggers an innate immune response that involves recognition by Toll-like receptor 4 (TLR4), which stimulates both myeloid differentiation primary response 88 (MyD88) dependent and MyD88-independent pathways that then activate the nuclear factor κB pathway and results in the release of pro-inflammatory cytokines. Interestingly, TLRs seem to have a role in pancreatic cancer development, and TLR4 is explicitly highly expressed in human pancreatic cancer but not a healthy pancreas. In the murine models, the TLR4/MyD88 pathway can trigger protection from pancreatic cancer development or acting to promote inflammation and pancreatic cancer development (63). LPS was shown to drive pancreatic carcinogenesis, as was the blockade of the MyD88-dependent pathway (via a dendritic cell-mediated deviation to T_H_2), whereas blockade of TLR4 (via TRIF, TIR-domain-containing adapter-inducing interferon-β) and blockade of the MyD88 independent (via TRIF) were protective against pancreatic cancer (63). Although more empirical evidence is needed, similar mechanisms implicating inflammatory response of the innate immune system may be implicated in other types of cancer.

### Oral Microorganisms and Cancers Outside the Oral Cavity

One characteristic of the oral microbiome that distinguishes from other body sites is that many oral microorganisms that are considered commensals in the oral cavity are commonly associated with various cancers in distant organs (**Table 1**). The associations of oral organisms and distant cancers occur in two categories. The first appears in certain types of cancer where no oral microbes are directly involved in the tumor’s pathogenesis, but there is a consistent change in the composition of the oral microbiome associated with cancer, thus with the potential use of those changes as biomarkers of cancer (71, 86). The second kind of association is the association of organisms involved in tumorigenesis, such as *F. nucleatum* association with CRC (88, 89, 100).

**Table 1:** Oral organisms associated with distant tumors.

Cancer	Organisms	Sample type	Reference
Esophageal cancer	Increase of *T. forsythia* and *P. gingivalis*	Oral rinse	(64)
Esophageal cancer	*Streptococcus anginosus*, *S. mitis*, *Treponema denticola*	Saliva	(65)
Esophageal cancer	3 taxon model: *Lautropia*, *Streptococcus*, and an unspecified genus of the order *Bacteroidales.* (AUC = 0.94)	Oral swab	(66)
Esophageal cancer	Overall decreased microbial diversity in cancer patients	Saliva	(67)
Pancreatic cancer	*Porphyromonas gingivalis, Aggregatibacteractinomycetemcomitans*	Oral rinse	(68)
Pancreatic cancer	*Porphyromonas gingivalis*	Blood (antibodies)	(69)
Pancreatic cancer	*Fusobacterium* spp.	Tissue from pancreatic ductal adenocarcinoma	(70)
Pancreatic cancer	2 taxon model: *Streptococcus mitis* and *Neisseria elongata.* (AUC =0.90)	Saliva	(71)
Pancreatic cancer	Significative higher ratio of *Leptotrichia*to *Porphyromonas* was found in cancer patients.	Saliva	(72)
Pancreatic cancer	Association with β-diversity and *Haemophilus*	Saliva	(73)
Pancreatic cancer	*Fusobacterium* spp.	Tissue samples, swabs, stool	(74)
Pancreatic cancer	*Streptococcus thermophilus* higher in cancer*, and Haemophilus parainfluenzae* and *Neisseria flavescens* lower in cancer	Saliva	(75)
Pancreatic cancer	*Haemophilus, Porphyromonas, Leptotrichia* and *Fusobacterium* could distinguish cancer patients from healthy subjects	Tongue coating microbiota	(76)
Hepatic cancer	*Fusobacterium* and *Oribacterium*. Increase in diversity.	Tongue coat	(77)
Lung cancer	*Capnocytophaga* sp., *Veillonella* sp.	Saliva	(78)
Lung cancer	*Streptococcus* and *Veillonella*	Airway brushings	(79)
Lung cancer	*Sphingomonas* and *Blastomonas*	Saliva	(80)
Lung cancer	*Streptococcus* and *Veillonella*	Saliva	(81)
Colorectal cancer	*T. denticola* and *Prevotella* sp. oral taxon 313	Oral rinse	(82)
Colorectal cancer	*Fusobacterium* sp., *Porphyromonas* sp.	Stool	(83)
Colorectal cancer	*Fusobacterium sp.*	Colorectal cancer tissues	(84)
Colorectal cancer	*Lactobacillus* and *Rothia*	Oral rinse	(85)
Colorectal cancer	*Streptococcus* and *Prevotella* spp.	Oral swabcolonic mucosae and stools, colorectal polyps or controls	(86)
Colorectal cancer	*Fusobacterium sp.*	Tissue and stool samples	(87)
Colorectal cancer	*Fusobacterium nucleatum*	Colorectal tissue biopsies	(88)
Colorectal cancer	*Fusobacterium sp.*	Colorectal tissue biopsies	(89)
Colorectal cancer	*Fusobacterium sp.*	Colorectal tissue biopsies	(21)
Colorectal cancer	*Fusobacterium sp., Lactococcus sp.*	Colorectal tissue biopsies	(90)
Digestive tract cancer	*Actinomyces odontolyticus, Steptococcus parasinguinis, Corynebacterium* spp*., Neisseria* spp.,TM7[G-1] sp., *Porphyromonas gingivalis, Fusobacterium nucleatum, Neisseria elongata and Streptococcus sanguinis*	Saliva	(91)
Colorectal cancer	*Fusobacterium nucleatum, Parvimonas micra*, and *Peptostreptococcus stomatis*	Colon tissue	(92)
Colorectal cancer	*Peptostreptococcus stomatis, Fusobacteriumnucleatum, Parvimonas* spp.	Meta-analysisfecal samples	(93)
Gastric cancer	Overall diversity of tongue coating microbiota was reduced	Tongue coating	(94)
Gastric cancer	Overall increased microbial diversity in cancer patients	Saliva and plaque samples	(95)
Gastric cancer	6 bacterial clusters were identified to distinguish cancer patients from controls. (cluster 6 had AUC = 0.76)	Tongue coating	(96)
Breast cancer	*Corynebacterium, Staphylococcus, Actinomyces*, and *Propionibacteriaceae*	Urine	(97)
Breast cancer	*Fusobacterium, Atopobium, Gluconacetobacter, Hydrogenophaga* and *Lactobacillus*	Breast tissue	(98)
Breast cancer	*Coriobacteriaceae*	Oral rinse	(99)

Dysbiosis of the oral microbiome has been associated with a wide variety of cancers (**Table 1**). There are three types of associations between microbiome and disease that have been described as potential biomarkers in cancer: increase or decrease in numbers of individual organisms, use of models of several organisms as predictors and assessing their performance, and finally changes in diversity indexes that give an overall picture of the behavior of the community.

As examples of specific organisms as biomarkers, an increase in *F. nucleatum* has been linked to CRC (88), and *P. gingivalis* (69) and *Fusobacterium* spp. (70) have been linked to pancreatic cancer. Additional examples of links of specific bacteria and oral organisms are presented in **Table 1**. Another approach in the search for biomarkers of cancer is the use of complex models of several organisms that maximize the area under the Receiver Operating Characteristics (ROC) curve or Area Under Curve (AUC). ROC shows how well a model can discriminate or separate the cases and controls, and AUC has a value between 0.50 and 1.0, indicating no discrimination and perfect discrimination, respectively (101). Thus, and AUC of 0.9 would indicate that the probability of discriminating a case vs. control is 90%. In **Table 1**, we show three examples of combined taxons to distinguish controls vs. cancer samples: a three taxon model of *Lautropia*, *Streptococcus* and an unspecified genus of the order *Bacteroidales*, with AUC =0.94, has been associated with esophageal cancer (66), a two taxon model, *Streptococcus mitis* and *Neisseria elongata* with an AUC of 0.90, associated with pancreatic cancer (71) and a bacterial cluster associated with gastric cancer with AUC of 0.76 (96). Finally, another approach to the use of biomarkers in differentiating case vs. control samples is using changes in biodiversity indicators as a proxy of changes in the microbiome that could be linked to the status of the sample, independently of what organisms are present or absent. We have also presented examples of this approach in **Table 1**. For instance, Chen et al. observed an overall decrease of microbial diversity in the saliva of esophageal cancer patients (67), while an overall increase in diversity in saliva and plaque from gastric cancer patients was observed by Sun et al. (95).

Except for CRC, where *Fusobacterium* spp. are ubiquitous, in general, there is little overlapping in the biomarkers identified in the different studies (**Table 1**), probably due to the different type of samples and methods of analysis used to identify the composition of the community.

Pancreatic cancer has been one of the most widely studied types of cancer in seeking association between changes in the oral microbiome and disease (68–76). Although the communities vary depending on the study, *Porphyromonas* and *Fusobacterium* genera were associated with cancer in most studies (**Table 1**). Likewise, in the case of lung cancer, the genus *Veillonella* appeared associated with the majority of studies (**Table 1**).

CRC has been an exception in that *Fusobacterium* spp. have been consistently associated with this type of cancer, both as a biomarker in the oral cavity (91) and more importantly, in stool and biopsies from tumors samples (21, 84, 87–90, 92, 93), indicating a possible direct effect on the progression of the disease. A more direct indication of the clinical relevance of *F. nucleatum* in CRC has been presented in a recent paper where the authors show that in mice with colorectal tumors, oral or intravenous administration of dextran nanoparticles covalently linked to azide-modified phages that inhibit the growth of *F. nucleatum* significantly augments the efficiency of first-line chemotherapy treatments of CRC (102). For all these reasons, most focus has been placed on studying the role of *F. nucleatum* in CRC. Using arbitrarily primed PCR (AP-PCR) to identify isolates at the strain level, Komiya et al. demonstrated that the strains present in CRC samples were identical to strains isolated from the saliva of CRC patients, supporting the oral origin of *F. nucleatum* in the intestine of CRC patients (103).

In 2012 Kostic et al., using genomic analysis, identified *Fusobacterium* sequences as enriched in colon carcinomas; they did not prove a causal relationship between *Fusobacterium* and colorectal cancer was the first indication of its potential importance in CRC (21). One year later, Kostic et al., using the mouse model of intestinal tumorigenesis Apc(Min/+), showed that *F. nucleatum* was capable of increasing tumor multiplicity and selectively recruit tumor-infiltrating myeloid cells, which can promote tumor progression. Tumors from Apc(Min/+) mice exposed to *F. nucleatum* exhibit a pro-inflammatory expression signature shared with human fusobacteria-positive colorectal carcinomas. *F. nucleatum* generates a pro-inflammatory microenvironment conducive to colorectal neoplasia progression by recruiting tumor-infiltrating immune cells (87). This same year Rubinstein et al. proposed a possible mechanism by which *F. nucleatum* induces tumorigenesis (104). *F. nucleatum* encodes several adhesins for interspecies interactions, but so far, only one, FadA (adhesion protein FadA), has been identified as binding to host cells (105). FadA is not only an adhesin but also an invasin required for binding and invasion of both healthy and cancerous host cells and binds to cell-junction molecules, the cadherins (105). In Rubinstein et al. model, FadA binds to E-cadherin activating β-catenin signaling (104). The activation of β-catenin signaling in colorectal cancer is mediated *via* a TLR4/P-PAK1 (p21-activated kinases) cascade (106). Loss of E-cadherin-mediated-adhesion characterizes benign lesions’ transition to invasive, metastatic cancer (107, 108).

In a more recent article, Rubinstein et al. expanded their model to include Annexin A1, a previously unrecognized modulator of Wnt/β-catenin signaling, which is a crucial component through which *F. nucleatum* exerts its stimulatory effect. Annexin A1 is expressed explicitly in proliferating colorectal cancer cells and involved in the activation of Cyclin D1 (109). Over-expression of cyclin D1 has been linked to the development and progression of cancer (110). Based on their results, Rubinstein et al. proposed a “two-hit” model to explain how *F. nucleatum* acts in CRC. Somatic mutations cause the first “hit”, and the second “hit” is caused by *F. nucleatum*, exacerbating cancer progression on those cells that suffered the initial mutations (109).

### The Microbiome and Oral Cancer

Head and neck cancer was the seventh most common cancer worldwide in 2018 (890,000 new cases and 450,000 deaths) (111). Oral squamous cell carcinomas (OSCC), the most frequent malignancies in the oral cavity, represented 2% of all cancers worldwide (354,864 cases and 177,384 deaths) (111). In the United States alone, it is expected that a total of 52,260 new cases of oral and pharynx cancer will occur in the year 2020 (112). The financial cost of treating oral and oropharyngeal cancer may be the highest of all cancers in the United States (113). Additionally, an essential factor in OSCC mortality is the high level of recurrence after treatment. Several studies, with many cases, have shown that the overall recurrence rate was approximately 30% (114–116). Recurrence rate in OSCC is high. In the first 36 months, the recurrence rates range from 70 to 92% of cases (114, 117–119). Interestingly the observed 5-year survival after recurrence varies depending on the moment in which recurrence occurs. If recurrence occurs within the 18 months after treatment, the rate of survival ranges between 20.5 to 27.55%, but if recurrence occurs afterward, the rate of survival increases to 38.1% to 42.3% (114, 120).

Despite advances in our knowledge of the causes and risk factors associated with OSCC, survival rates for oral cancers, including OSCC have not improved substantially in the last forty years, emphasizing that new means of early detection and treatment are urgently needed.

Viruses have long been associated with the risk of developing OSCC. The etiological role of human papillomavirus (HPV) in OSCC has been widely researched for more than three decades. Several meta-analyses have demonstrated that infection by HPV increments the risk of OSCC by up to 3-fold (121, 122). The prevalence of HPV among OSCC patients is around 25% (121, 123, 124); however, there is a geographical component with the highest HPV prevalence in Africa and Asia, notably among Chinese studies from provinces with high OSCC incidence rates (123). Other viruses have been identified in OSCC samples, as individual infections or in co-infection with HPV. Still, their role in the disease is unclear (124–127). Despite its importance, HPV-unrelated OSCC cases account for the vast majority of oral cancer cases. Other environmental factors should play an essential role in OSCC development and progression; among them, the microbiome’s role has just begun to be considered a risk factor.

As mentioned previously, periodontitis has been linked to various types of cancers, including esophagus/oropharyngeal cancers. **Table 2** presents some of the studies that linked bacteria’s presence to cancer of the oral cavity and pharynx. Several studies have found that the risk of developing OSCC may increase with periodontal disease (55, 142, 143), signaling a possible role of inflammation caused by the microbiome with oral cancer. Periodontitis is a typical example of an infectious disease causing chronic inflammation in the oral cavity (144, 145). Thus several systematic reviews of the literature showed that periodontal disease increases the risk of oral cancer even after adjusting for significant risk factors (146, 147).

**Table 2:** Oral organisms in oropharyngeal cancers.

Cancer	Organisms	Sample type	Reference
Head and neck squamous cell carcinoma (HNSCC)	*Streptococcus sp. and Lactobacillus sp.*	Saliva	(128)
HNSCC	*Streptococcus anginosus*	Tissue	(129)
HNSCC	*Fusobacterium sp.*	Meta-analysis	(38)
Oral squamous cell carcinoma (OSCC)	*Streptococcus anginosus*	Tissue	(130)
OSCC	*Capnocytophaga gingivalis, Prevotella melaninogenica, Streptococcus mitis*	Saliva	(131)
OSCC	*Bacillus, Enterococcus, Parvimonas,Peptostreptococcus, Slackia*	Saliva	(132)
OSCC	*Streptococcus sp. 058, S. salivarius, S. gordonii, S. parasanguinis, Peptostreptococcus stomatis, Gemella haemolysans, G.morbillorum, Johnsonella ignava*	Tissue	(133)
OSCC	*Parvimonas* increased in OSCC, *Actinomyces* reduced in OSCC	Tissue	(134)
OSCC	*Fusobacterium periodonticum, Parvimonas micra, Streptococcus constellatus, Haemophilus influenza*, and *Filifactor alocis*	Oral rinse	(80)
OSCC	*Fusobacterium nucleatum, Prevotella intermedia, Aggregatibactersegnis, Capnocytophaga leadbetteri, Campylobacter rectus,Catonella morbi, Corynebacterium matruchotii, Gemella morbillorun, Granulicatella adjacens, Granullicatella elegans, Peptococcus* sp.*, Peptostreptococcus stomatis, Porphyromonas catoniae* and *Streptococcus oralis*	Tissue	(135)
OSCC	*Fusobacterium, Dialister, Peptostreptococcus, Filifactor, Peptococcus, Catonella* and *Parvimonas*	Swabs	(136)
OSCC	*Micrococcus luteus, Prevotella melaninogenica, Exiguobacterium oxidotolerans, Fusobacterium naviforme, Staphylococcus aureus, Veillonella parvula, Prevotella sp. (oral clone BE073 phylotype), Rothia mucilaginosa, Streptococcus salivarius, Actinomyces odontolyticus, Moraxella osloensis, Prevotella veroralis, Propionibacterium acnes, Atopobium parvulum, Streptococcus parasanguinis, Veillonella dispar, Streptococcus mitis/oralis*	Tissue	(137)
Gingival squamous cell carcinoma	*P. gingivalis*	Paraffin embedded samples	(138)
Oral mucosal cancer	*Streptococcus intermedius, S. constellatus, S. oralis, S. mitis, S. sanguis, S. salivarius, Peptostreptococcus sp.*	Lymph nodes	(139)
Keratinizing squamous cell carcinoma	*Veillonella* sp., *Fusobacterium* sp.*, Prevotella* sp.*, Porphyromonas* sp., *Actinomyces* sp.*, Clostridium* sp*., Haemophilus* sp*., Streptococcus sp.*, and *Enterobacteriaceae*	Swabs	(140)
Potentially malignant oral leukoplakia	*Fusobacterium, Leptotrichia, Campylobacter* and *Rothia*	Swabs	(141)

Moreover, expression of pro-inflammatory cytokines in periodontal disease such as IL-1 and TNF-α has been linked to microbial triggered carcinogenesis (19). In a study comparing the microbiome of gingival squamous cell carcinoma (GSCC) with periodontitis microbiome, members of the genera *Fusobacterium*, *Peptostreptococcus*, and *Prevotella* were more abundant in cancerous, periodontal tissues. In contrast, saliva or soft mucosa harbored more periodontal health-related bacteria (148).

Most studies on the role of the human microbiome on cancer have focused on describing microbial communities present in specific samples or the immunological response of the host to the bacterial challenge. The oral microbiome has been proposed as a diagnostic indicator of oral cancer; however, as in other types of cancer, the search for possible biomarkers of oral cancer and, most specifically, OSCC has not produced conclusive results (52). Different organisms have been shown to increase in OSCC samples, as a few examples: *Capnocytophaga gingivalis*, *Prevotella melaninogenica*, and *S. mitis* (131); *F. nucleatum* (149); *Pseudomonas aeruginosa* (150); *Campylobacter concisus*, *Prevotella salivae*, *Prevotella loeschii*, and *Fusobacterium* oral taxon 204 (151); genera *Fusobacterium*, *Dialister*, *Peptostreptococcus*, *Filifactor*, *Peptococcus*, *Catonella*, and *Parvimonas* (136); and *Prevotella oris*, *Neisseria flava*, *Neisseria flavescens/subflava*, *F. nucleatum ss polymorphum*, *Aggregatibacter segnis*, and *Fusobacterium periodonticum* (152).

Yang et al. studied the progression of the microbiome during cancer’s progression from the early to the late stage and found a significant increase of *Fusobacteria.* At the species level, they found that *F. periodonticum*, *Parvimonas micra*, *Streptococcus constellatus*, *Haemophilus influenza*, and *Filifactor alocis* were associated with OSCC, and they progressively increased in abundance from stage 1 to 4 (80).

Direct evidence of the role of the microbiome in OSCC was presented by Stashenko et al. were using a germ-free mouse model a 4-nitroquinoline-1 oxide (4NQO)-induced carcinogenesis the authors observed a significant increase on the number of tumors and their size when the mice were inoculated with two different microbiomes vs. inoculated controls. The microbiomes used came from the tongue of a healthy mouse and the other from the tumor lesion of a diseased mouse, and *Pasteurella* was the dominant genus in both groups (153).

The carcinogenic potential of periodontal pathogens has also been described in several studies. *P. gingivalis*, one of the essential periodontal pathogens, increase the invasiveness of oral cancer cells and resistance to chemotherapeutic agents (154–156). Using a 4NQO-induced mouse model of oral cancer, the inoculation of *P. gingivalis* promoted tumor progression by invading precancerous lesions and recruiting the myeloid-derived suppressor cells by expressing chemokines such as C-C motif ligand 2 (CCL2) and chemokine (C-X-C motif) ligand 2 (CXCL2), and cytokines such as IL-6 and IL-8 (157). *P. gingivalis* also induces expression of the ZEB1 transcription factor, which controls the epithelial-mesenchymal transition. Interestingly, the up-regulation of ZEB1 appears to be controlled by FimA, a major virulence factor involved in adhesion and cellular invasion (158). The infection of *P. gingivalis* increases the expression of mesenchymal markers, including vimentin and matrix metalloproteinase MMP-9 (132, 158, 159).

Utilizing a murine model of periodontitis-associated oral tumorigenesis, Binder Gallimidi et al. showed that chronic bacterial co-infection of *P. gingivalis* and *F. nucleatum* promotes OSCC *via* direct interaction with oral epithelial cells through Toll-like receptors, with an increase in expression of TLR2 in OSCC cells and IL-6 in both cells and the mouse model (160). A detailed review of all the studies where *P. gingivalis* has been associated with the development of OSCC has been recently published by Lafuente Ibáñez de Mendoza et al. (161).

*F. nucleatum* in esophageal cancer tissues has been associated with shorter survival, suggesting a prognostic biomarker’s potential role. *F. nucleatum* might also contribute to aggressive tumor behavior through the activation of chemokines, such as chemokine (C-C motif) ligand 20 (CCL20) (162). Epithelial-mesenchymal transition (EMT) is the process by which epithelial cells acquire a mesenchymal-like phenotype. It has been proposed that EMT is responsible for compromising epithelial barrier function in the pathogenesis of several diseases, including OSCC. *F. nucleatum* infection of oral cell lines triggers EMT (163, 164). *F. nucleatum* triggers EMT *via* lncRNA/MIR4435-2HG/miR-296-5p/Akt2/SNAI1 signaling pathway and up-regulates mesenchymal markers, including N-cadherin, Vimentin, and snail family transcription repressor 1 (SNAI1) (164). The fact that infection by periodontal pathogens generates EMT features introduces the possibility that this process may be involved in the loss of epithelial integrity during periodontitis and may promote predisposition to malignant transformation through the EMT.

The metatranscriptome has recently begun to be used to analyze community-wide gene expression in the human microbiome (165–168). Measuring bacterial gene expression in the wild has been challenging. The half-life of mRNA is short, and mRNA in bacteria and archaea usually comprises only a small fraction of total RNA. Therefore large samples are needed to study expression, but obtaining such samples is not always possible. Additionally, working with non-model organisms presents the challenge of lacking most of the bioinformatic tools readily available for model organisms.

Using a metatranscriptome analysis of OSCC samples, Yost et al. found that *Fusobacteria* showed a statistically significantly higher number of transcripts at tumor sites, indicating a higher activity of this group of organisms in cancer. Moreover, when looking at tumor signatures of the oral microbiome, metabolic activities such as iron ion transport, tryptophanase activity, peptidase activities, and superoxide dismutase were over-represented in tumor samples when compared to the healthy controls (169).

### Effect of the Microbiome in the Outcomes of Cancer Therapy

Despite its clinical importance, the human microbiome’s effect on malignancy treatment is merely starting to be investigated. While organisms assume significant activities in keeping up human well-being, they are likewise engaged with the turn of events that lead to tumor development. There is now proof indicating that the microbiome can impact patient reactions to cancer treatment. The microbiome has been implicated in modulating cancer therapy’s efficacy and toxicity, including chemotherapy and immunotherapy (170, 171). Moreover, preclinical data suggest that the microbiome modulation could become a novel strategy for improving the efficacy of immune-based therapies for cancer (172).

The gut microbiota has the potential to affect the ability of cancer therapy. The microbiota, when affected by dysbiosis, can profoundly influence both cancer pathogenesis and its therapeutic outcome. In particular, the regulation of such a therapeutic outcome is firmly linked with the gut microbiota’s capacity to process anti-cancer compounds and modify the host’s immune response. These two effects combined may clarify the patient’s microbiome composition’s substantial participation in affecting the efficiency of both immunotherapy and chemotherapy.

The oral microbiome’s role in the outcome of cancer treatments has not yet been evaluated. Only in two studies, oral organisms were identified as influencing the outcome of immunotherapies. The first of these studies showed that *F. nucleatum*, along with *Bacteroides fragilis* and *Escherichia coli* improved the survival of adoptive cell therapy (ACT) treated patients, probably by increasing cytokine production and T cell infiltration (173). The second study found that *Lactobacillus fermentum* attenuated the immune response in patients treated with CpG-oligonucleotides, which are short synthetic single-stranded DNA molecules containing unmethylated CpG dinucleotides, acting as agonists of Toll-like receptor 9 (TLR9), and leading to strong immunostimulatory effects (174).

## Effects On Cancer Chemotherapy

A recent survey of *in situ* bacterial effects on frequently used chemotherapeutics suggests the profound influence of distinct bacteria species on the anti-tumor effect. Heshiki et al. examined the influence of the intestinal microbiome on treatment effects (175) in a heterogeneous cohort that comprised eight diverse malignancy types to recognize organisms with a collective effect on the immune response. It is revealed through human gut metagenomic examination that responder patients had inherently higher microbial diversity arrangements than non-responders. Moreover, by assessing the gut microbiome’s job without precedent for a heterogeneous patient cohort with different kinds of malignant growth and anti-cancer medicines, Heshiki et al. found a worldwide microbiome signature that is autonomous of disease type and heterogeneity (175). Explicit species, *Bacteroides xylanisolvens*, and *Bacteroides ovatus* were decidedly connected with treatment results. Oral gavage of these responder microbes fundamentally expanded the adequacy of chemotherapy (erlotinib) and actuated the declaration of CXCL9 and IFN-γ in a murine lung cancer model. Also, oral gavage of explicit gut microbiome substantially expanded the impact of chemotherapy in mice, decreasing the tumor volume by 46% contrasted with the control.

This information recommends an anticipated effect of the microbiome’s explicit constituents on tumor development and disease treatment results with suggestions for both visualization and treatment. Curiously, the microbiome can bolster the resistant framework in the battle against malignancy. For instance, cyclophosphamide (a medication used to treat leukemia and lymphomas) was found to impact the organisms living in the gut. These gut organisms reacted by advancing the production of resistant cells, which appears to improve cyclophosphamide adequacy (176). Among patients with hematologic malignancies, specific bacterial taxa are connected with the effectiveness of allogeneic hematopoietic stem cell transplantation (allo-HSCT) and reduced hazard for graft-versus-host disease (GVHD) succeeding treatment (177, 178). Besides, holding expanded measures of microorganisms having a place with the variety of *Blautia* was related to diminished GVHD lethality in this cohort and was affirmed in another independent cohort of 51 patients from a similar organization (177).

## Effects On Cancer Radiotherapy

Ionizing radiation therapy (RTX) was received by cancer patients that are genotoxic for tumor cells and might be curative for restricted cancers. The great worldview in radiation biology accepted that the cellular nucleus was the main objective of radiation and DNA harm was incited by direct testimony of vitality or creation of reactive oxygen species (ROS) through a radiation-induced separation of intracellular water molecules. Ionizing radiation, however, additionally incites non-targeted impact on non-irradiated cells, such as genomic unpredictability, systemic radio-adaptive retorts, inflammatory and immune reactivity, and bystander effect on nearby cells. Observer and foundational impacts are auxiliary to DNA harm and are intervened by the interruption of gap intersection proteins engaged with cell-cell interactions and by the arrival of extracellular intermediaries, together with cytokines, exosomes, ROS and nitric oxide (NO). Along these lines, comparably to the tissue harm related to contamination by microorganisms, radiations initiate the arrival of damage-associated molecular pattern (DAMP) pressure signals (179). The impacts of radiation are perplexing. It initiates both immunostimulant and immunosuppressive reactions and might be deficient in enacting a defensive anti-cancer invulnerable reaction (180). It very well may be speculated that the gut microbiome likewise satisfies a job in the immunostimulatory impacts of RTX. The viewed fluctuations in microbiome arrangement at epithelial surfaces in patients and mice rewarded with RTX have been proposed to contribute to the pathogenesis of bone marrow failure, colitis, looseness of the bowels, oral mucositis and enteritis (181). RTX persuades apoptosis in the breach of the intestinal barrier, intestinal crypts, and modifications in the microbiome conformation (182).

Studies have shown that the intestinal microbiome has a significant effect on total body irradiation. Fewer endothelial cells of the intestinal mucosa are derived through irradiation into apoptosis and prompt less lymphocyte invasion in germ-free mice than in conventional mice (183). This finding shows that gut commensals can assume a harmful role in protecting the enteric harmfulness of total body irradiation (TBI) in germ-free mice.

## Effects On Cancer Immunotherapy

Ongoing examinations have featured the significance and possible effect of organisms on ailment recuperation of immunotherapy. Strikingly, the microbiome can bolster the safe framework in the battle against malignant growth (176). Microorganisms have been appeared to advance cancer growth improvement by instigating inflammation. This provocative reaction can, likewise, beneficially affect malignancy treatment. A few treatments, such as CpG-oligonucleotide immunotherapy, rely on inflammation (174).

In an examination performed by Iida et al., it was seen that mice treated with anti-toxins did not react to platinum chemotherapy or CpG-oligonucleotide insusceptible treatment contrasted with mice with flawless gut microorganisms of subcutaneous tumors. These outcomes recommend that gut microbiome improves the impacts of treatments that are subject to aggravation. Desirable reactions to cancer therapy require an unblemished commensal microbiome that intervenes in its belongings by regulating myeloid-inferred cell capacities in the tumor microenvironment. These discoveries underscore the significance of the microbiome in the outcomes of sickness treatment (174).

In most patients treated with traditional cancer therapies, tumors become resistant to therapy, and the chances of tumor recurrence are high (184). Immunotherapy approaches have shown potential in treating hematopoietic (185, 186) and solid cancers (187–189). However, the efficacy of immunotherapy is still limited by the variability of the immune response in different patients and the different susceptibility of tumor types (190, 191). The emerging knowledge of the ability of the gut microbiome to modulate the response to immunotherapy offers new possibilities to improve its efficacy by targeting the microbiome.

Utilization of Check Point Inhibitors (ICIs) has revolutionized cancer treatment across multiple cancer types and has gotten the first since forever FDA endorsement of a tumor agonistic agent in tumors with microsatellite instability (MSI) (192). The most generally utilized ICIs are monoclonal antibodies that focus on the customized cell demise protein (PD-1), its ligand (PD-L1), or the cytotoxic T-lymphocyte antigen 4 protein (CTLA-4). **Table 3** shows the microbiome in malignant growth patients dealt with immune checkpoint inhibitors (ICIs). It was proposed by Sivan (197) that *Bifidobacterium*, a particular taxon of microbial commensals, armed anti-tumor resistance and raised the viability of PD-L1 blocking treatment. They additionally recommended that microbiome could modify the anti-tumor invulnerability as well as the reaction to PD-L1 inhibitors. Routy et al. investigated the relationship of dysbiosis with epithelial tumors to comprehend whether synchronous utilization of anti-infection agents creates essential protection from ICIs in mice and patients. Their outcomes indicated that the anti-tumor impact was undermined in antibiotic (ATB) treatment group, with progression-free survival (PFS) and overall survival (OS), being fundamentally shorter contrasted with that of the control group, showing that ATB could be utilized as a prescient marker for estimating ICIs obstruction. Additionally, utilizing the shotgun sequencing for quantitative metagenomics of the fecal example, *Enterococcus hirae* and *Akkermansia muciniphila* were demonstrated to be altogether plentiful in patients with best clinical reaction to ICIs (PFS > 3 months) (196).

**Table 3:** Characteristics of the microbiome in cancer patients treated with immune check point inhibitors (ICIs).

Cancer	Microbes related with response to ICI treatment	References
Metastatic melanoma	*B. fragilis* and/or *B. thetaiotaomicron, Burkholderiales* species	(193)
Metastatic melanoma	*Faecalibacterium* genus and other Firmicutes	(194)
Metastatic melanoma	*Enterococcus faecium*, *Collinsella aerofaciens*, *Bifidobacterium adolescentis*, *Klebsiella pneumoniae*, *Veillonella parvula, Parabacteroides merdae*, *Lactobacillus* species, and *Bifidobacterium longum*	(195)
Non-small cell lung carcinoma	*Ruminococcaceae*, *Faecalibacterium*, specifically *Ruminococcae, Alistipes*, and *Eubacterium* species	(196)
Renal cell carcinoma	*Ruminococcaceae*, *Faecalibacterium*, specifically *Ruminococcae, Alistipes*, and *Eubacterium* species	(196)

Naidoo et al. established a connection between the gut microbiome and remedial results after the clinical examination of Chinese patients with cutting edge non-little cell lung carcinoma treated with PD-1 ICIs treatments. As indicated by their outcomes, reacting patients held greater assorted variety and stable arrangement of the natural gut microbiome during treatment and had drawn-out PFS altogether. In detail, *Prevotella copri*, *Bifidobacterium longum*, and *Alistipes putredinis* were improved in responders, though *Ruminococcus* spp. was found mainly in non-reacting patients. As expected, in the periphery blood of responding patients, a more noteworthy recurrence of natural killer cell and memory CD8+ T cell subgroups was seen (198).

A consortium of 11 bacterial strains was isolated by Tanoue et al. from healthy human donor feces that can thrive in the intestine, increasing interferon-γ-producing CD8 T cells. The colonization of mice improved immune checkpoint inhibitors’ therapeutic effectiveness with these 11-strain mixtures in syngeneic tumor models. All these strains act together in a way that is subject to significant histocompatibility (MHC) class Ia molecules and CD103^+^ dendritic cells. Primarily the 11 strains represent rare, low-abundance components of the human microbiome, and subsequently, have incredible potential as extensively powerful biotherapeutics (199).

Then again, immunotherapy viability has all the earmarks of being intensely impacted by gut microbiome confirmation. Oral organization of probiotics, for example, *A. muciniphila* (196) and *Bifidobacterium* species (197) or fecal microbiota transplantation (FMT) (200) from treatment-responsive patients, considerably upgraded the PD1-based immunotherapy and canceled tumor outgrowth, robotically through the enlarged dendritic cell and T cell reaction (197). Even though these examinations are not utilizing colorectal cancer (CRC) models, seeing how gut microbiome adjusts strong reaction might be necessary to encourage positive remedial results in CRC patients getting immunotherapy, or even to conquer opposition-held by non-responders. To our best information, no clinical preliminaries assessing gut microbiome control and treatment viability are distributed right now (201). A couple of clinical preliminaries are started and now at the selecting stage (**Table 4**) in light of Fong et al. (201). It stays dark whether these preclinical discoveries can be effectively meant clinical application.

**Table 4:** Ongoing clinical trials of gut microbiota modulation in potentiating efficacy of anticancer therapies.

Patient /Cancer	Number of subjects	Intervention	Primary outcomes	Secondary outcome	Location	Status	Clinical trial registration number
**Chemotherapy**							
Patients with metastatic CRC	50	Chemotherapy + Weileshu (*Lactobacillus salivarius* AP-32, *Lactobacillus* *johnsonii* MH-68) vs chemotherapy alone	PFS	OS	Zhejiang, China	Not yet recruiting	NCT04021589
Patients with metastatic CRC	140	Chemotherapy + targeted therapy + Bifico (*Lactobacillus acidophilus* and *Bifidobacterium*) vs chemotherapy + targeted therapy	ORR	/	Zhejiang, China	Not yet recruiting	NCT04131803
Rectal cancer patients receiving concurrent chemotherapy and pelvic Radiation therapy	160	VSL#3 vs placebo	Impact of probiotics to increase tumor regression grade (TRG) 1-2 rate	Acute bowel toxicity Pathological complete response Sphincter saving surgery Disease-free survival Late toxicity (at 12-36 months)	Rome, Italy	Recruiting	NCT01579591
**Immunotherapy**							
Melanoma patients resistant/refractory to PD-1 therapy	20	Single-arm: FMT from anti-PD1 responders through colonoscopy + PD-1 therapy	ORR	T cell composition T cell function Immune profile	Pennsylvania, United States	Recruiting	NCT03341143
Patients with solid tumors (including non-small cell lung cancer, renal cell carcinoma, bladder cancer or melanoma	132	Single-arm: MRx0518 + Pembrolizumab	Adverse events	Tumor biomarkers Clinical benefits (ORR, DOR, DCR, PFS) Microbiome composition OS	Texas, United States	Recruiting	NCT03637803

PFS, progression-free survival; OS, overall survival; ORR, objective response rate; DOR, duration of response; DCR, disease control rate.

The microbiome, aside from the balance of adequacy, may likewise foresee the susceptibility to immunotherapy-associated unfavorable events. While ICIs have given way to overawed immunological resilience to tumors, the danger of auto insusceptibility in healthy tissues is a critical restriction in their utilization. In patients on anti-CTLA-4, the immune-related adverse events (irAEs) happen all the more generally contrasted with those taking anti-PD-1/PD-L1, and when these mediators are used in amalgamation, the frequency of irAEs appears to upsurge consequently (198). The microbiome has been related to the danger of creating immune-related noxiousness. The abundance of species according to study by Dubin et al. (202) among Bacteroidetes phylum, explicitly *Barnesiellaceae*, *Bacteroidaceae*, and *Rikenellaceae*, was related with resistance to colitis in patients with metastatic melanoma dealt with ipilimumab (*n*=34), though reduced recognition of hereditary pathways engaged with polyamine transport and vitamin B amalgamation in the gut related with an expanded danger of colitis (202).

Moreover, Chaput et al. showed that baseline gut microbiota is a good predictor of clinical response and colitis in metastatic melanoma patients treated with ipilimumab. Twenty-six patients with metastatic melanoma treated with ipilimumab were enrolled in the study. Fecal microbiota composition was assessed at baseline and before each ipilimumab infusion. In their results, baseline gut microbiota enriched with *Faecalibacterium* and other Firmicutes was associated with beneficial clinical response compared with patients whose baseline microbiota was driven by *Bacteroides* (194).

The significant reason for repeat and poor prognosis is the treatment failure in colorectal cancer patients, resistance to malignancy medicines has been connected to the nearness of explicit sorts of microbes in the gut. In colorectal cancer patients, scientists observed that resistance to drugs correlated with an expansion in *F. nucleatum* in the gut. The bacterium appeared to square passing (apoptosis) of the malignant growth cells and trigger autophagy, an endurance device for the disease cells. Yu et al. explored the commitment of gut microbiome to chemoresistance in patients with colorectal malignant growth (203). In colorectal cancer tissues in patients with relapse post-chemotherapy, the *F. nucleatum* was found in abundance and was related to patient clinicopathological qualities. They additionally showed that *F. nucleatum* elevated colorectal malignant growth protection from chemotherapy. *F. nucleatum*, mechanistically, targets TLR4 and MYD88 inborn immune signaling and specific microRNAs to enact the autophagy pathway and modify colorectal malignant growth chemotherapeutic reaction. To control colorectal cancer chemoresistance mechanistically, clinically, and biologically, the F. nucleatum arranges an atomic system of the Toll-like receptor, autophagy, and micro-RNAs. Estimating and focusing on *F. nucleatum* and its related pathway will yield critical understanding into clinical administration and may enhance colorectal malignant patient outcomes (203).

Albeit a progression of examinations has confirmed the effect of the microbiome on disease treatment, there still lies a lot of vagueness and deficiency in these investigations. The initial test experienced is the inadequate comprehension of the rare microbial species engaged with a better strong reaction.

### The Potential of Microbiome in Cancer Therapy

The worldwide malignant growth trouble has risen drastically, making it a critical need to create novel treatments and anticipate which treatment will offer the most advantage to a disease quiet. The use of microorganisms to treat tumors is nothing new. German physician W Busch probably treated the first patient with cancer to be purposefully infected with bacteria in 1868 (204). He induced a bacterial infection in a woman with an inoperable sarcoma by first cauterizing the tumor and then placing her into bedding previously occupied by a patient with “erysipelas” (*Streptococcus pyogenes*). Busch reported that within a week, the primary tumor and the lymph nodes in the neck had shrunk in size. Unfortunately, the patient died a few days after the infection had begun. Later, in 1883 Friedrich Fehleisen, a German surgeon had identified *S. pyogenes* as the cause of “erysipelas” and had begun treating patients with cancer with the living cultures of the bacteria with success (205). Almost simultaneously in the USA, William B. Coley was performing similar experiments. In 1981 he injected *Streptococcus* into a patient with inoperable cancer (206). The infection caused by the bacterium has the side effect of shrinking the malignant tumor, and this was one of the first examples of immunotherapy. Given the risks of using live organisms, Coley developed a mixture of dead bacteria to treat his patients. They were known as Coley’s toxins. He then devoted most of his life as head of the Bone Tumor Service at Memorial Hospital in New York, treating more than 1000 cancer patients with bacteria or bacterial products (207). However, his studies, the first using immunotherapy, were forgotten for several reasons. First, the use of microorganism(s) to treat human cancers provided a short term benefit, but eventually, tumors recurred; second, the discovery of radioactivity and its use to treat tumors in the early 1930s and second the advent of chemotherapy in the 1940s relegated the use of immunotherapies on a second plane.

In recent years, there has been a renewed interest in using bacteria-mediated tumor therapy (204, 208, 209). Although widely used for treating many tumors, chemotherapy causes many off-target effects, such as significant damage to healthy tissues. In contrast, biological generally exert target-specific effects and are relatively safer for human use. Moreover, bacterial-based immunotherapies can penetrate solid tumors and inexpensive. Upon systemic administration, various types of non-pathogenic obligate anaerobes and facultative anaerobes have been shown to infiltrate and selectively replicate within solid tumors (204, 209).

A significant step in the development of bacterial therapeutics is identifying potential species and strains with minimal pathogenicity to the host, and that can replicate precisely in the tumor hypoxic microenvironment. Several genera of bacteria, including *Clostridium*, *Bifidobacterium*, *E. coli*, and attenuated *Salmonella*, have been used in bacteria-mediated tumor therapy (204, 208–210).

The genera *Clostridium* and *Salmonella* are probable the best-studied as vectors in bacteria-mediated tumor therapy. In 1947, it was first shown that direct injection of spores of *Clostridium* *histolyticus* into a transplantable mouse sarcoma caused oncolysis and tumor regression; however, mice died soon after (211). In 1964 Moese and Moese injected a non-pathogenic isolate of *Clostridium butyricum* intravenously and observed the tumor’s disappearance, but again the survival of the mice was non-permanent (212). The ability of *Clostridium* to grow in hypoxic areas with necrosis gives them an advantage to target tumors. With the development of genetic systems to work with *Clostridium*, this bacterium has attracted renewed interest as a potential vector to treat solid tumors. Thus, a genetically engineered *C. acetobutylicum* expressing and secreting the *E. coli* cytosine deaminase (CDase) has been used against rhabdomyosarcoma-bearing WAG/Rij rats and show that the enzyme was produced in tumors *in vivo* (213).

Numerous *S. typhimurium* mutant strains have been studied from the perspective of cancer treatment. One of the significant advantages of using *S. typhimurium* is that there is a well-developed genetic system that allows for all kinds of genetic manipulations of this organism. Many different immunomodulators have been cloned into *S. typhimurium* to be expressed at the tumor site. These include cytotoxic proteins, to kill tumor cells directly, and tumor-associated antigens to increase the immune response in the site of the tumor, and prodrug enzymes that modify a substrate to convert it into a toxic product (214, 215).

So far, the only strain of *S. typhimurium* going through a phase I clinical trial is strain VNP20009, which contains deletions in the *msb*B and *pur*l genes, to attenuate virulence and avoid septic shock (216).

A more recent approach has been combining bacteria-mediated cancer therapies with other kinds of therapies. For example, a photo-thermal agent, such as melanin-like poly-dopamine (pDA), was coated with VNP20009 targeted to hypoxic and necrotic tumor areas. A mouse model of the tumor was irradiated with a near-infrared laser, which achieved tumor targeting and tumor elimination without relapse or metastasis (217).

Although no reports on the treatment of OSCC have been published, the potential of these techniques to treat oral tumors is enormous. OSCC are solid tumors, and they are easily accessible to be treated at the site, which makes the ideal type of cancer treated with bacterial-mediated tumor therapies.

## The Future of Microbiome in Cancer Therapy

We anticipate that the human microbiome will bit by bit play an undeniably conspicuous role in cancer treatment. As of now, the system of the microbiome’s impacts in cancer treatment is not surely known; be that as it may, some clinical pilot studies will assist with uncovering the capability of the microbiome in tumor advancement and malignancy treatment. The advancement of these clinical preliminaries will expel impediments for utilizing the microbiome to improve and help treatment utilizing ICIs. The microbiome, at first, can lessen complexities during malignant growth treatment. Right now, the most widely recognized noxious reaction when utilizing ICIs is related to colitis. The reason for the ailment is uncertain. Curiously, the lactic acid bacterium *Lactobacillus reuteri* can wipe out ICIs related to colitis and improve weight reduction and irritation (218). Second, the intestinal microbiome upgrades the nourishing assimilation limit of patients with cancer and improves their anti-tumor capacity. The rise of tumor micro-ecological immune nutrition has additionally prepared for the improvement of the microbiome as implement in disease immunotherapy. Third, microbiome research is relied upon to prompt the structure of immunization against tumors. An ongoing microbial-based malignancy immunization has demonstrated its utility. This malignant growth antibody forestalls the development of squamous cell carcinoma communicating epidermal growth factor receptor (EGFR) vIII and instigates EGFR vIII-explicit cell insusceptibility (219).

Current research is thrilling for investigation against tumor resistance and signifies a discovery in the structure of the microbiome. For cancer immunotherapy, FMT is predictable to be the most critical immediate bio optimization tool. FMT is a mainstream innovation that has been utilized clinically to treat repetitive *Clostridium difficile* contaminations (220).

Finally, recent studies support the hypothesis that periodontal inflammation exacerbates gut inflammation *in vivo* by translocation of oral pathobionts to the gut, activating the inflammasome in colonic mononuclear phagocytes resulting in inflammation (221, 222). Additionally, periodontitis results in the generation of reactive Th17 cells against oral bacteria. These reactive Th17 cells are exhibit gut tropism and migrate to the inflamed gut. When in the gut, Th17 cells of oral origin can be activated by translocated oral pathobionts and cause the development of colitis, but gut-resident microbes do not activate them. We do not know if gut inflammation could have a similar effect on the oral cavity’s inflammatory environment, as a result of which the severity of head and neck cancer could increase. Studies of the gut microbiome’s effect on the immune response to head and neck cancer are lacking but maybe worth pursuing.

## Author Contributions

All authors contributed equally to the writing of the review. All authors contributed to the article and approved the submitted version.

## Funding

National Institute of Dental and Craniofacial Research. Grant DE021553.

## Conflict of Interest

The authors declare that the research was conducted in the absence of any commercial or financial relationships that could be construed as a potential conflict of interest.
